# Bioengineered Extracellular Vesicles Mitigate Neuroinflammation by Neutralizing Pneumolysin and Delaying Disease Onset in Experimental Pneumococcal Meningitis

**DOI:** 10.1002/jev2.70369

**Published:** 2026-09-11

**Authors:** Kristine Farmen, Doste R. Mamand, Miguel Tofiño‐Vian, Georgia Yfanti, Xiuming Liang, Houze Zhou, Vicky W. Q. Hou, Samir El Andaloussi, Oscar P. B. Wiklander, Federico Iovino

**Affiliations:** ^1^ Department of Neuroscience Karolinska Institutet Stockholm Sweden; ^2^ Division of Biomolecular and Cellular Medicine, Department of Laboratory Medicine Karolinska Institutet Stockholm Sweden; ^3^ Karolinska ATMP Center Karolinska Institutet Stockholm Sweden; ^4^ Breast Center Karolinska Comprehensive Cancer Center Karolinska University Hospital Stockholm Sweden; ^5^ Biology Research Center, Research Center University of Zakho Zakho, Duhok Kurdistan Region Iraq; ^6^ Department of Cellular Therapy and Allogeneic Stem Cell Transplantation (CAST) Karolinska University Hospital Stockholm Sweden

**Keywords:** *Streptococcus pneumoniae*, pneumococcal meningitis, bioengineered extracellular vesicles, neuroinflammation, neuronal protection, RVG, IL‐6, pneumolysin

## Abstract

Bacterial meningitis is a life‐threatening neurological disorder frequently caused by a *Streptococcus pneumoniae* (the pneumococcus) infection of the brain. Standard treatment consists of antibiotics to eliminate bacteria and dexamethasone to reduce inflammation. Despite this, mortality reaches 20% in treated individuals, and half of the survivors suffer long‐term neurological sequelae. This is largely due to the poor capacity of antibiotics to reach the brain and the lack of antimicrobial treatment capable of neutralizing the pneumococcal toxin pneumolysin (Ply). To address these limitations, we isolated extracellular vesicles (EVs) derived from human HEK293T cells and evaluated their therapeutic potential in pneumococcal meningitis. Alongside wild‐type EVs (WT.EVs), we bioengineered EVs to express RVG peptides (RVG.EV) for targeting neuronal acetylcholine receptors, signal incompetent IL‐6 signal transducer (IL‐6ST) decoy receptors (IL‐6.EV) to block the pro‐inflammatory signalling of IL‐6, or EVs expressing both RVG peptides and IL‐6ST (DB.EV). *In vitro*, all EVs reduced pneumococcal adhesion to neurons and mitigated cytotoxicity by binding and sequestering Ply. In a bacteremia‐derived pneumococcal meningitis model, EV treatment significantly increased the survival of the mice without affecting bacterial load in the brain or the periphery. Among all groups, RVG.EV treatment was most effective in reducing pro‐inflammatory cytokine release in the periphery and brain. These findings highlight the therapeutic potential of bioengineered EVs, particularly RVG peptides expressing EVs, as an adjunctive treatment for pneumococcal meningitis thanks to their (i) sequestration and neutralization of Ply, (ii) increased blood‐brain barrier crossing, and (iii) dampening of inflammation.

## 1 Introduction

*Streptococcus pneumoniae* (the pneumococcus) infections are responsible for one million deaths of children worldwide annually (WHO 2017). The most serious manifestation of the disease is invasive pneumococcal disease, where the bacterium enters sterile body sites such as the blood and brain, causing bacteremia and meningitis, respectively (Weiser et al. 2018). *S. pneumoniae* is a major cause of bacterial meningitis worldwide and is associated with a worse patient prognosis (Van De Beek et al. 2016, Sharew et al. 2020). Survival is dependent on rapid access to treatment; however, even among treated individuals, a mortality rate of 20% is observed, and about 50% of survivors experience permanent neurological sequelae due to neuronal damage inflicted by bacteria and bacterial toxic components (Engelen‐Lee et al. 2016, Generoso et al. 2022). This poor prognosis is driven by challenges with antibiotic treatment, including antibiotic resistance, the low ability of antibiotics to cross the blood‐brain barrier (BBB), which leaves the brain untreated for a prolonged period, and exacerbated neuroinflammation due to bacterial lysis by antibiotics (Van De Beek et al. 2012, Nau et al. 2015).

*S. pneumoniae* activates inflammatory cascades, leading to the release of pro‐inflammatory cytokines and chemokines (Loughran et al. 2019). Elevated TNF‐α and IL‐6 levels in the cerebrospinal fluid (CSF) correlate with disease severity in patients, and the release of chemokines increases the infiltration of peripheral monocytes and neutrophils to aid microglia in the clearance of the infection; however, the clearance is inefficient, and a prolonged inflammatory state occurs (Mook‐Kanamori et al. 2011, Grandgirard et al. 2013). This is detrimental as inflammatory components such as matrix metalloproteinases and reactive oxidative species are toxic to cells (Gerber and Nau 2010). The upheld neuroinflammation is associated with increased intracranial pressure, alteration in blood flow, herniation, and infarction (Engelen‐Lee et al. 2016; Wall et al. 2021). Dexamethasone is used to mitigate neuroinflammation and has been shown to improve patient outcomes (Fritz et al. 2012).

In addition to the host‐mediated damage, *S. pneumoniae* causes cellular damage by releasing pneumolysin (Ply) (Farmen et al. 2021). Ply is a potent stimulator of pro‐inflammatory cytokines through interaction with Toll‐like receptor (TLR) 2 and 4 on immune cells (Mitchell and Dalziel 2014). The toxin also aids *S. pneumoniae* evasion from microglial cells by reducing their motility (Hupp et al. 2019). Ply is a cholesterol‐dependent cytolysin, and the interaction with membrane cholesterol generates a pore in the cell membrane, causing cell lysis (Shewell et al. 2014; Tilley et al. 2005; Serra et al. 2024). In addition, Ply facilitates the binding of *S. pneumoniae* to neuronal cells, disrupting the cytoskeleton, leading to cell death (Tabusi et al. 2021; Serra et al. 2024). Further, it contributes to hearing loss and cortical damage in experimental pneumococcal meningitis (Winter et al. 1997; Reiß et al. 2011). Ply also disrupts the ciliary beating in ependymal cells and causes cytoskeleton remodeling in astrocytes, potentially affecting the CSF flow in the brain and contributing to brain edema (Hirst et al. 2000; Hupp et al. 2012). Importantly, increased Ply concentrations in the brain correlate with increased risk of death in patients (Wall et al. 2012).

Since the cellular damage observed in pneumococcal meningitis is a consequence of the neuroinflammatory process and direct damage by *S. pneumoniae*, novel therapies must effectively and quickly reach the brain, reduce *S. pneumoniae* cytotoxicity towards cells by blocking direct interaction, neutralize Ply, and reduce inflammation. Extracellular vesicles (EVs) have the potential to fulfil all of these requirements thanks to their intrinsic beneficial properties; they exhibit low immunogenicity and are rich in cholesterol and thus putatively bind Ply (Pfrieger and Vitale 2018). Furthermore, EVs cross cellular barriers, including the BBB, the latter is increased during systemic inflammation (Morad et al. 2019; Banks et al. 2020). Importantly, they can be bioengineered to express receptors and peptides (Du et al. 2023).

We focused on three such strategies: (1) Bioengineering of EVs with rabies viral glycoprotein (RVG) peptides that bind to the nicotinic acetylcholine (nACh) receptors widely expressed by neurons (Huey et al. 2017; Abbondanza et al. 2024). This expression of RVG was reported to cause a two‐fold increase in brain invasion compared to wild‐type EVs (Huey et al. 2017; Wiklander et al. 2015). Thus, we hypothesized that EVs expressing RVG peptides could act as a molecular barrier between neurons and *S. pneumoniae*, reducing the cellular cytotoxicity during infection. (2) Bioengineering EVs expressing signal incompetent IL‐6 signal transducer (IL‐6ST) decoy receptors that block the pro‐inflammatory trans‐signalling pathway of IL‐6 (Gupta et al. 2021). (3) Generating double‐coated EVs expressing both RVG peptide and IL‐6ST, hypothesizing that this inhibition would lower both neurotoxicity and inflammation. We also speculated that the EVs could, due to their membrane cholesterol, bind Ply and thus reduce cellular cytotoxicity. Therefore, we assessed the therapeutic potential of wild‐type EVs (WT.EV) or bioengineered EVs expressing either RVG peptides (RVG.EV) or signal incompetent IL‐6ST decoy receptors (IL‐6.EV) or both RVG.IL‐6EVs (DB.EV) from HEK293T cells in an experimental pneumococcal meningitis model.

*In vitro*, we demonstrated that all EVs sequestered Ply and reduced *S. pneumoniae*‐induced neuronal cytotoxicity during infection. Further, by inhibiting trans‐signalling of IL‐6, the release of pro‐inflammatory cytokines was reduced. In a bacteremia‐derived meningitis mouse model, all EV types had a protective effect and delayed disease onset. Interestingly, the RVG.EV treatment outperformed the rest of the treatment groups and reduced both microglial activation and release of pro‐inflammatory cytokines in the brain and periphery. Ultimately, we demonstrated that treatment with bioengineered EVs expressing RVG peptides in pneumococcal meningitis was effective and holds vast potential as an adjuvant treatment in severe pneumococcal infections.

## 2 Materials and Methods

### 2.1 EV Production and Isolation

In total four different EV types were generated: wild‐type EVs (WT.EV), the single expressing EVs RVG.EV and IL‐6.EV, containing the RVG peptides and signal incompetent IL‐6ST decoy receptors respectively, and finally the double‐expressing EVs containing both RVG peptides and the signal incompetent IL‐6ST decoy receptors (DB.EVs). The EVs were generated as previously described (Wiklander et al. 2015; Gupta et al. 2021; Wiklander et al. 2024). Briefly, EVs were generated through polyethylenimine (PEI)‐mediated transient transfection of HEK293T cells. Cells were initially seeded in 15‐cm dishes at a density of 10 × 10^6^ cells per dish using complete DMEM (10 % FBS and 1% penicillin‐streptomycin‐antimycotic) (Gibco,). After 48 h, cells were transfected with the appropriate plasmids. Six hours post‐transfection, the culture medium was replaced with Opti‐MEM (Gibco) supplemented with 1% penicillin‐streptomycin‐antimycotic. After an additional 48 h, the conditioned medium (CM) was harvested and purified by sequential centrifugation at 700 × g for 5 min and 2000 × g for 10 min. The resulting supernatant was passed through a 0.22 µm filter to remove cellular debris and large particles.

EVs were isolated from a filtered CM using tangential flow filtration (TFF) using a MicroKross system (20 cm^2^, Spectrum Labs) equipped with a 300 kDa molecular weight cutoff membrane, allowing for selective retention and concentration of vesicle‐sized particles. The retentate was further concentrated using Amicon Ultra‐15 centrifugal filters (100 kDa cutoff, Millipore) by centrifugation at 4000 × g at 4°C. Spin durations ranged from 30 min to several hours, depending on the sample volume and EV content. The resulting EV concentrate was transferred into maximum recovery 1.5 mL microtubes (Axygene,) and analyzed by Nanoparticle Tracking Analysis to determine particle concentration and size distribution.

### 2.2 EV Characterization

#### 2.2.1 Nanoparticle Tracking Analysis (NTA)

NTA measurements were conducted with the Zetaview system (Particle Metrix). All samples were diluted in 0,22 µm filtered PBS to achieve a final volume of 1 mL. The ideal measurement concentrations were determined through pre‐testing to achieve a particle count of 140–200 particles per frame. The manufacturer's default software settings for EVs were selected accordingly. In brief, the settings included a sensitivity of 80 and a shutter speed of 100. Two cycles were performed for each measurement, scanning 11 cell positions and capturing 60 frames per position (with the video setting set to high). The specific settings for the measurements were as follows: Focus was set to autofocus; camera sensitivity was set to 80.0; shutter speed remained at 100; scattering intensity was adjusted to 4.0; and the cell temperature was maintained at 24°C. After video capture, the data were analyzed using the built‐in ZetaView Software version 8.02.31.

#### 2.2.2 Western Blotting

The intravesicular protein levels of TG101, surface RVG and Gp130 (IL‐6ST) in engineered EVs were determined using western blot analysis, as previously described (Mamand et al. 2024). Calnexin was used as a marker for cell lysates and to assess the purity of EVs. In brief, 2 × 10^10^ EVs and 2 × 10^6^ HEK293T cells were lysed in 100 µL of radioimmunoprecipitation buffer (RIPA; BioRad, Hercules). The samples were incubated on ice for 30 min, vortexed for 10 s every 5 min. Afterward, the EVs and cell lysates were mixed with 8 µL of loading buffer (10% glycerol, 8% sodium dodecyl sulfate, 0.5 M dithiothreitol, and 0.4 M sodium carbonate). This mixture was incubated at 65°C for 5 min, loaded onto a NuPAGE+ (Invitrogen, Novex 4%–12% Bis‐Tris gel), and run at 120 V for 1.5 h. The proteins were transferred to an iBlot membrane (iBlot 2 Transfer Stacks; Invitrogen) using the iBlot 2 Dry Blotting System for 7 min. The membrane was then incubated with blocking buffer (Odyssey Blocking Buffer; LI‐COR Biosciences) at room temperature (RT) for 1 h and subsequently incubated overnight at 4°C with freshly prepared primary antibodies: Anti‐RVG (1:1000, Invitrogen, Cat#:PA5‐117507), anti‐TSG101 (1:500, Invitrogen, Cat#:MA1‐23296) anti‐calnexin (1:000, Invitrogen Cat#: MCA497G), and anti‐Gp130 (IL‐6ST) (1:1000, Invitrogen, Cat#14‐5982‐82). The membranes were washed four times with TBS‐T 0.1% for 5 min each time on a shaker. Following the washes, the membranes were incubated for 60 min with a 1:10000 diluted secondary antibody (IRDye 800CW Goat anti‐Mouse IgG, Goat anti‐Rabbit IgG and Donkey anti‐Goat IgG, LI‐COR Biosciences). The membranes were washed three times with PBS, and the results were visualized using an infrared imaging system (LI‐COR Odyssey CLx).

The protein levels of albumin, to address possible serum contaminants in EV preparation, and Ply, CD63, and CD81 used to assess EV‐Ply binding and EV brain tissue infiltration were performed as previously described (Serra et al. 2024). Briefly, samples were resuspended in LDS sample buffer (1:4, Invitrogen) and boiled at 95°C for 10 min and loaded onto NuPage+ (Invitrogen, Novex 4%–12% Bis‐Tris gel). Electroblotting was performed using the Mini Gel Tank (Thermo Fisher Scientific). Samples were blocked with PBS‐T (T = 0,1% Tween) supplemented with 5% skim‐milk powder (Merck) for 1 h at RT; and incubated with primary antibodies: Albumin (1:1000, Invitrogen), Ply (1:1000 Abcam), CD63 (1:1000, Abcam), CD81 (1:1000, Invitrogen) overnight. Membranes were washed in PBS‐T and incubated with secondary antibodies (1:5000, Goat anti‐Rabbit IgG (H+L), Invitrogen) for 1 h. Both primary and secondary antibodies were diluted in PBS‐T supplemented with 2.5% skim‐milk powder (Merck). Protein bands were detected by incubating membranes with ECL Prime Western Blotting Detection Reagents (Cytiva) and imaged using ImageQuant LAS 4000 (GE Healthcare). For Coomassie staining, gels were incubated with InstantBlue Coomassie Protein Stain (Abcam) overnight and imaged. Band intensities were measured using the analyze gels plug‐in and the area under the curve (AUC) using ImageJ (Fiji). For ultracentrifugation experiments, the total Ply AUC was divided by the total AUC for Coomassie staining, for qEV Ply experiment the Ply AUC was divided by the CD63 AUC. The sequestering capacity of the EVs was calculated as Pellet Ply/ Total Ply (Pellet + supernatant fraction).

#### 2.2.3 Single‐Vesicle Imaging Flow Cytometry for Characterization of EV Surface Markers

Characterization of EV surface markers using flow cytometry was done as previously described (Abbondanza et al. 2024). Shortly, purified EVs were diluted to a concentration of 1 × 10^10^ particles/mL using Dulbecco's Phosphate‐Buffered Saline. A total of 2.5 × 10^8^ EVs in 25 µL were stained with either 8 nM of phycoerythrin (PE), fluorescein isothiocyanate (FITC), or Allophycocyanin (APC) conjugated antibodies targeting the surface markers or proteins of interest, RVG‐APC (Novus Biologicals), and Gp130‐APC (IL‐6) (eBioscience), CD9‐PE (Miltenyi Biotec), CD63‐PE (Miltenyi Biotec), or CD81‐FITC (Miltenyi Biotec), which are known to be enriched in EVs. The EVs were then incubated overnight at room temperature in the dark within a sealed 96‐well plate (SARSTEDT AG & Co. KG). After incubation, the samples were diluted in a 1% human albumin and trehalose (PBS‐HAT) buffer solution to achieve a final concentration of 1 × 10^7^ EVs/mL in a total volume of 100 µL (Wiklander et al. 2015). Single‐vesicle imaging was performed using an Amnis Cellstream (Luminex) and the results examined in FlowJo (v. 10.8; FlowJo LLC).

#### 2.2.4 Transmission Electron Microscopy

3µL of each EV sample was applied to glow‐discharged, carbon‐coated, formvar‐stabilized 400 mesh copper grids (easiGlow, Ted Pella) and incubated for approximately 30 sec. The excess sample was blotted off, followed by a MilliQ water wash. Grids were negatively stained with 1% ammonium molybdate and imaged using a HT7800‐Xarosa transmission electron microscope (Hitachi High‐Technologies) operated at 80 kV, equipped with a 4 MP Veleta CCD camera (Olympus Soft Imaging Solutions GmbH).

### 2.3 Cultivation of *Streptococcus pneumoniae*

*Streptococcus pneumoniae* TIGR4 (serotype 4) (T4) was grown to exponential phase (OD of 0.3) in Todd‐Hewitt broth (THY, Substrat, KI) and stored in THY solution supplemented with 20% glycerol (Substrat, KI) at −80°C. Before *in vitro* experiments, the bacterial aliquots were thawed, pelleted, and washed in PBS to remove any trace of glycerol, and resuspended in cell culture media. The day before the *in vivo* experiments, the aliquots were thawed, pelleted, resuspended in PBS, and frozen overnight at −20°C.

### 2.4 Cell Culture

Human SH‐SY5Y neuroblastoma cells were cultured as previously described (Tabusi et al. 2021). Briefly, cells were seeded in 6‐well plates at a density of 300.000 cells/well, or 100.000 cells/well in 12‐well plates, differentiated for 12 days to obtain mature neuronal cells using growth media made of equal parts EMEM (ATCC) and F12 (Gibco), 5% heat inactivated fetal bovine serum (FBS) (Gibco), 1% penicillin/streptomycin (Gibco), and 10 µM retinoic acid (RA) (Biotechne). Differentiation into mature neuronal cell was assessed via detection of the neuronal marker MAP2 (monoclonal anti‐MAP2 antibody, Abcam) through western blot analysis. The BV‐2 mouse microglial cell line (Cytion, Eppelheim,) was plated at a density of 300.000 cells/well in 6‐well plates in DMEM medium (Gibco) supplemented with 2.5% heat‐inactivated FBS (Gibco) and 1% penicillin/streptomycin (Gibco). All cell lines were incubated at 37°C with 5% CO_2_. Media was changed every 2^nd^ or 3^rd^ day. One day before infection experiments, the cells were changed to their respective growth media without antibiotics. HMC3 human microglial cells (ATCC CRL‐3304) were cultured as previously described (Farmen et al. 2026). Briefly, cells were maintained in EMEM supplemented with 10% FBS and penicillin–streptomycin at 37°C and 5% CO_2_. Prior to infection, medium was replaced with antibiotic‐free DMEM containing 2.5% FBS; for experiments with corticosteroid treatment, bethametasone (Evolan Pharma) was used at the concentration of 1 µM (Chantong et al. 2012) 30 min before infection.

### 2.5 *In Vitro* Bacterial Adhesion and Cytotoxicity Assays

*In vitro* assays for bacterial adhesion and cytotoxicity were performed as previously described (Serra et al. 2024; Farmen and Tofiño‐Vian 2023). Shortly, on the day of the experiment, the cell medium was discarded, and cells were washed in pre‐warmed PBS and changed to pre‐warmed cell medium without RA for SH‐SY5Y and antibiotics for both cell lines. The cells were incubated for 1 h at 37°C in 5% CO_2_ to acclimatize. Cells were infected with a Multiplicity of Infection (MOI) = 10 for neuronal cells and 20 for BV‐2 cells and treated with 1 × 10^11^ p/mL of EVs or equal volumes of PBS (control) at the same time as infection (EV: Cell ratio = 10^6^:1) or added 30 min post infection (EV: Cell ratio = 10000:1). In assays with antibiotic treatment, a dosage of 25 µg/mL Ceftriaxone (Navamedic) was added 30 min post‐infection. Infected cells were incubated at 37°C and 5% CO_2_ for 2 h, subsequently, the supernatants were collected (non‐adhered bacteria), and cells were washed with PBS to eliminate unbound bacteria. Next, cells were treated with 1 mL of trypsin for 15 min, and the cell suspension was collected (adhered bacteria). Serial dilutions were performed of the non‐adhered and adhered fractions and plated on blood agar plates overnight. The following day the Colony Forming Units (CFUs) were determined. The percentages of adhered bacteria were determined as the (adhered bacteria)/(non‐adhered + adhered bacteria) × 100%.

For the cytotoxicity assays, the CyQUANT LDH Cytotoxicity Assay Kit (Invitrogen, Cat#C20300 and C20301) was used to measure the released lactase dehydrogenase (LDH), as an assessment of cytotoxicity. Infection was performed as previously described, while for assessment of Ply sequestering and cytotoxicity, SH‐SY5Y neuronal cells were treated with 2.0 µg/mL recombinant Ply (Protein Production Platform, Nanyang Technological University, Singapore), in combination with 1 × 10^9^ p/mL of EV groups, controls were treated with PBS for 45 min (EV:Cell ratio = 10000:1). After infection/treatment, 50 µL from the supernatant of each sample was transferred to a 96‐well flat‐bottom plate, in technical triplicates. Untreated and uninfected cells were used as negative controls; the positive controls were cells treated with lysate buffer (Invitrogen). Absorbance was measured with Multiskan FC microplate reader (Thermo Fisher Scientific) at 450 nm and 620 nm; to determine LDH activity, 620 nm absorbance value (background from the instrument) was subtracted to 450 nm absorbance value.

### 2.6 Scanning Electron Microscopy (SEM)

SH‐SY5Y‐differentiated neuronal cells were grown on glass coverslips (Fisher Scientific) and infected with T4 at MOI = 50 and treated with 2,3 × 10^11^ p/mL of DB.EVs for 30 min at 37°C with 5% CO_2_; untreated cells were used as a negative control (EV: Cell ratio = 10^6^:1). The cells were fixed by immersion in 2.5 % glutaraldehyde (Ladd Research) in 0.1 M phosphate buffer, pH 7.4. The coverslips were rinsed in phosphate buffer pH 7.4 followed by MilliQ water, followed by ethanol dehydration and critical point drying using carbon dioxide (Leica EM CPD 030). The coverslips were mounted on alumina SEM pins using carbon conductive tabs (Ted Pella) and sputter coated with a 10 nm layer of platina (Quorum Q150T ES). The images were acquired using an Ultra 55 field emission SEM (Zeiss) at 3 kV and the SE2 detector.

### 2.7 EV‐Ply Binding Assay

To assess the sequestering capacity of EVs, 2 µg/mL of Ply (Protein Production Platform, Nanyang Technological University, Singapore) was incubated with 2.2 × 10^9^ p/mL EVs for 30 min on ice in Ultra Clear Tubes (Beckmann Coulter) and placed in a precooled ultracentrifuge (Beckmann Coulter). The samples were centrifuged for 70 min at 100 000 × g at 4°C. The supernatant and pellet were collected separately, and BCA was performed to determine protein concentration prior to western blot analysis (described Section 2.2).

### 2.8 Size Exclusion Chromatography (SEC) for EV‐Ply Binding

70 nm qEV original columns (500 µL; IZON Science Ltd) were equilibrated according to the manufacturer's instructions. A mixture of 2.2 × 10^11^ p/mL of respective EV type and 2 µg/mL of Ply (Protein Production Platform, Nanyang Technological University, Singapore), pre‐mixed for 15 min RT, was loaded onto each column. The EV‐enriched fraction (4th and 5th mL eluted fractions) was collected, concentrated to a final volume of 200–300 µL using 2 mL 10 kDa molecular weight cut‐off spin filters (Amicon Ultra; Millipore) and centrifuged at 4000 × g at 4°C. The concentrated EVs were analyzed with NTA and the Ply concentration in each elute was determined by western blot analysis (described in Section 2.2)

### 2.9 Hemolytic Activity

For all hemolytic assays pooled human red blood cells (Medix biochemical) were washed by diluting 1:10 in PBS (Substrat) and centrifuged 500 × g 10 min at RT. The pellet was resuspended 1:100 in PBS. Hemolysis was induced by adding recombinant Ply (Protein Production Platform, Nanyang Technological University, Singapore) in the presence of EVs (concentrations are stated in figure legends). PBS alone was used as a negative control, and 0.03% Triton X‐100 (Merck) as a positive control. The solutions were placed on a shaking incubator at 37°C for 45 min, and non‐lysed red blood cells were pelleted by centrifugation 500 × g 10 min at RT. The supernatant was collected, and hemoglobin release was determined by absorbance measurement with Multiskan FC microplate reader (Thermo Fisher Scientific) at 450 nm. The percentage of hemolysis was calculated as follows: A_450_ sample‐OD A_450_negative control/ (A_450_positive control‐ A_450_negative control). All experiments were conducted with 3 technical replicates.

### 2.10 Animal Experiments

Five‐ to six‐week‐old male C57BL/6J mice (JAX, Charles River) were housed in a 13:11 h light/dark period with *ad libitum* access to water and food in accordance with Swedish legislation (Svenska jordbruksverket, ethical permit number 18965‐2021). We utilized a bacteremia‐derived meningitis model as previously described (Farmen et al. 2024; Vander Elst et al. 2025). For the first pilot study: Each mouse received an intravenous injection of 1 × 10^8^ CFU of *S. pneumoniae* in PBS solution through the tail vein, control animals received PBS. 1 h later, mice received treatment with 0.5–2 × 10^11^ particles/mouse WT.EVs through an I.V injection. Repeated intravenous tail vein injections separated by a 1 h interval were technically feasible in our experimental conditions, without observable re‐bleeding or leakage from the initial injection site during the second administration. Control animals received PBS. The mice were assessed for clinical symptoms every 3 h and scored according to Karolinska Institutet's ‘Assessment of health conditions of small rodents and rabbits when illness is suspected’ template. The experimental endpoint was set 8 h post‐T4 injection, to assess the effect of the EVs on bacterial growth *in vivo*. For the other animal experiments, the infection and treatment time points were the same, however, the treatment dosage was 2 × 10^11^ particles/ mouse for all EVs and the experimental endpoint was when the animal reached a score of 0.4–0.5, at which time the animals displayed both neurological and systemic symptoms of the disease. An overview of the different animal studies, the purpose, type of EV treatment, and downstream analysis is depicted in Table S1.

At the time of sacrifice, blood was collected for assessment of bacterial load and/or cytokine expression. The mice were perfused with ice‐cold PBS through the left ventricle, and the brain was collected and immediately dissected into two hemispheres. The left hemisphere was placed in ice‐cold 4% PFA (Histolab) for immunohistology downstream analysis, while the right hemisphere was placed in PBS for preparation of brain homogenates. The spleen was collected and placed in ice‐cold PBS. Spleen and brain tissue were homogenized through a 100 µm cell strainer and frozen at −80°C with 1% protease inhibitor cocktail added (Fisher Scientific). Serial dilutions of tissues and blood were plated on blood agar plates (Substrat) for the determination of CFU numbers and incubated overnight at 37°C with 5% CO_2_

### 2.11 Immunostaining and Confocal Microscopy

The left hemisphere of the brain was post‐fixed overnight at 4°C, placed in a 30% sucrose (Merck) solution for a minimum of 48 h at 4°C, and subsequently cut into coronal sections of 30 µm on a microtome (Leica SM2000). The sections were frozen in a cryoprotective solution (30% sucrose and 2% DMSO (Merck) in PBS) and stored at −20°C until further processing. Before staining, the sections were washed in PBS, blocked with 5% goat serum (Gibco), and permeabilized with 0.3% Triton‐X‐100 (Merck) for 1 h at RT and stained with primary antibodies overnight at 4°C. After washing with PBS, the sections were incubated with secondary antibodies for 2 h at RT. The primary antibodies used in this study were: Iba1 (1:1000 Abcam, Cat#:MA5‐36257), GFAP (1:250, Cell Signaling Technologies, Cat#:3670), and NeuN (1:250, Proteintech, Cat#:66836‐1‐Ig). Secondary antibodies used were Alexa Fluor conjugated anti‐immunoglobulin at 1:1000 (IgG Alexa Fluor 594, 488, Invitrogen, Cat#:A‐11001, A‐11012). The microvasculature was stained using Lectin‐594 conjugate (1:250, Vector Laboratories, Cat#:DL‐1177‐1). Images were obtained by taking a Z‐stack containing 20 µm thick sections. Images were taken at both 20 ×, 40 ×, and 63 × magnifications using a Zeiss LSM900‐Airy confocal microscope, with settings kept constant between respective image acquisitions used in quantification. For Iba1, NeuN, GFAP, and lectin staining, three coronal sections per mouse were analyzed.

### 2.12 *Ex Vivo* Analysis

#### 2.12.1 Enzyme‐Linked Immunosorbent Assay (ELISA)

Plasma was separated from whole blood by centrifugation at 1500 × g for 15 min at 4°C and together with supernatants from *in vitro* and brain and spleen homogenates from *in vivo* experiments stored at −80°C until analysis. The protein concentration was determined using the bicinchoninic acid assay (BCA) (Fisher Scientific), and equal protein concentrations were standardized by diluting samples in PBS. Quantification of IL‐6, TNF‐α, and IL‐10 was performed with the ELISA Mouse DuoSet kits (Bio‐Techne Cat#:DY406, #DY410, and #DY417) with a sensitivity of 15.6 ρg/mL for IL‐6 and 31.2 ρg/mL for IL‐10, and TNF‐α, respectively. Assays were performed following the manufacturer's instructions, loading a total of 100 µg of protein into each well in duplicate, and the optical density of each well was measured using a Multiscan FC microplate reader (Thermo Scientific) at 450 nm.

#### 2.12.2 Western Blot Analysis

To assess the protein levels of IL‐6 in spleen tissues, homogenates were mixed with LDS sample buffer (1:4, Invitrogen) and boiled at 95°C for 10 min and loaded into NuPage+ (Invitrogen, Novex 4%–12% Bis‐Tris gel). Electroblotting was performed using the Mini Gel Tank (Thermo Fisher Scientific). Samples were blocked with PBS‐T (T = 0,1% Tween) supplemented with 5% skim‐milk powder (Merck) for 1 h at RT and incubated with primary antibody for IL‐6 (1:1000, Abcam) overnight at 4oC. Membranes were washed in PBS‐T and incubated with the appropriate HRP‐conjugated secondary antibody goat anti‐Rabbit IgG (H+L) (1:5000, Invitrogen) for 2 to 3 h. All antibodies were diluted in PBS‐T supplemented with 2.5% skim‐milk powder (Merck). Protein bands were detected by incubating membranes with SuperSignal West Pico PLUS Chemiluminescent Substrate (Thermo Scientific) and imaged using ImageQuant LAS 4000 (GE Healthcare). Total protein staining was performed by incubating the gels overnight with PageBlue Protein Staining Solution (Thermo Scientific) followed by imaging with Gel Doc XR+ Gel Documentation System (Bio‐Rad). Densitometric analysis of the protein bands was performed in ImageJ using the Analyze Gels plug‐in and the area under the curve (AUC) using ImageJ (Fiji).

### 2.13 Image Processing and Analysis

ImageJ (Fiji) was used to analyse the confocal images. To assess the mean intensity and area coverage by Iba1 and GFAP, 40 × magnification stacks were merged, the background subtracted, and the image was converted to a binary image by threshold, and the area fraction was measured. GFAP images were taken at different anatomical locations in the brain, and to account for differences in cell density and morphology between regions, the data were normalized. Analysis of microglia morphology (process length and endpoints) was performed as previously described (Young and Morrison 2018). Briefly, an unsharp mask with a pixel radius of 3 and a mask weight of 0.6 value was used to increase contrast, followed by a despeckle step removing noise. The image was converted to a binary image by threshold and the steps despeckle, close‐ and removal of outliers (pixel radius 2, threshold 50) was applied to remove noise. The image was skeletonized using the skeleton plug‐in and parameters extracted with “analyze skeleton 2D/3D”. Short branches were removed with a cut of value of 5. Settings were kept constant between analyses. For NeuN, the images were thresholded and cells counted by the “analyzing particles” plug‐in with a cut size of 5 µm. Settings were kept constant between analyses. The total number of cells was divided by the total field of view (F.O.V) area.

### 2.14 Statistical Analysis

GraphPad Prism‐9 was used for statistical analyses. Outliers were identified using the ROUT test, *Q* = 1%. Normality was tested using the Shapiro–Wilk test. Ordinary one‐way ANOVA or Kruskal–Wallis test was performed using Bonferroni or Dunn's for multiple comparisons. Kaplan–Meier survival curves were analysed using a log‐rank (Mantel‐Cox) test. For comparison of *in vitro* ELISA data a two‐way ANOVA and a Bonferroni correction for multiple comparisons was performed. Information about statistical tests and the number of animals is specified in the respective figure legends.

## 3 Results

### 3.1 Extracellular Vesicles Alleviate Pneumococcal‐Induced Neuronal Cytotoxicity, and Blockade of IL‐6 Trans‐Signalling Mitigated Inflammation

**FIGURE 1:**
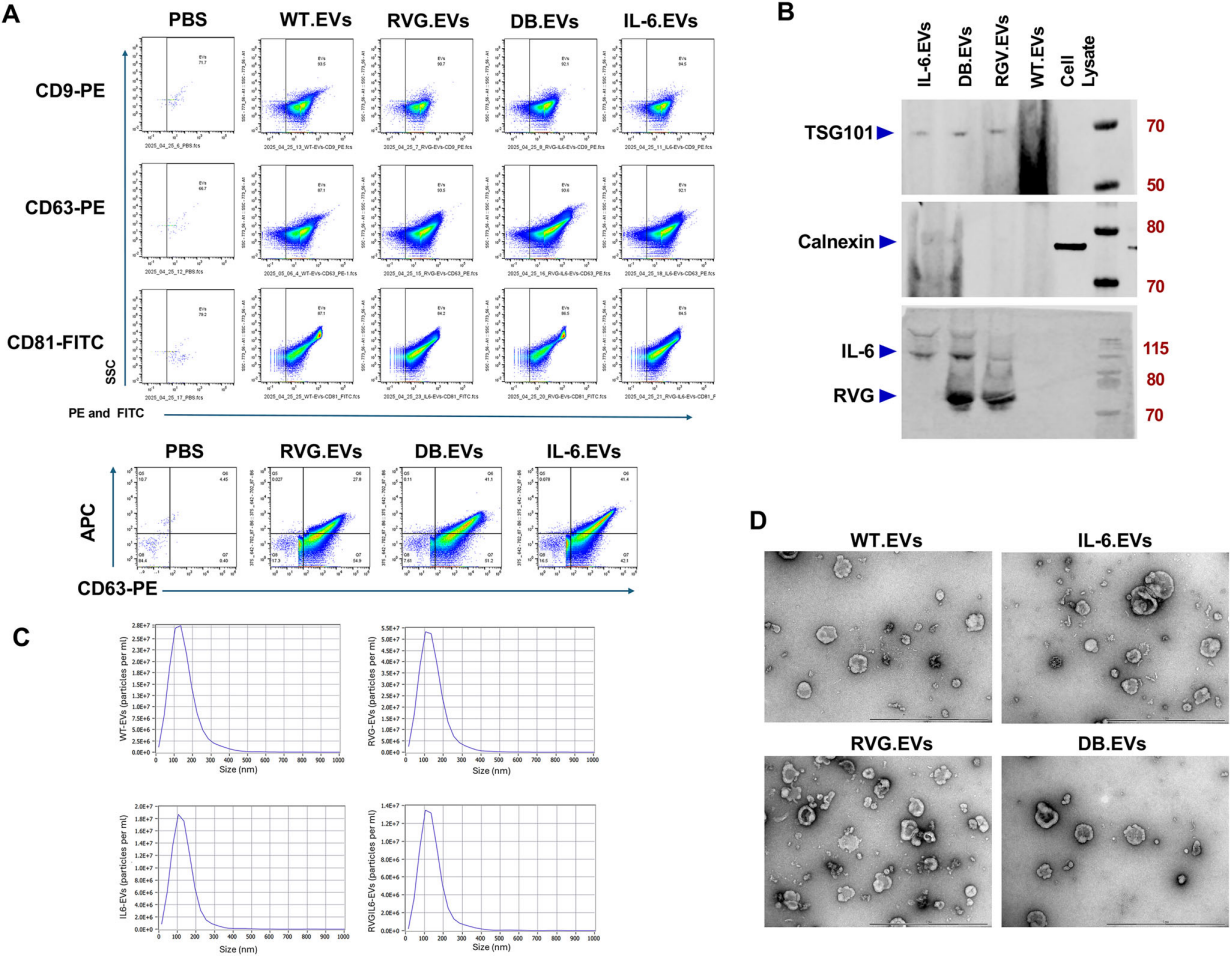
Characterization of WT.EVs and bioengineered EVs. The characterization of the EVs used in the *in vitro* and *in vivo* studies. (A) Flow cytometry analysis depicted in *upper panel*: similar expression of the tetraspanin EV markers CD9 CD63 CD81, between the EV types. *Lower panel*: confirmation of surface‐exposed RVG peptide (APC‐RVG) and IL‐6ST (APC‐IL‐6ST), in RVG.EVs, DB.EVs and IL‐6.EVs respectively. (B) Western blotting visualized the expression of IL‐6ST and RVG peptide for the engineered EVs. The intravascular protein TSG101 was present in all EVs, while calnexin was used as a cellular organelle marker. (C) NTA was performed to assess the size of the EVs for the WT.EVs, RVG.EVs, IL‐6.EVs, and RVG.IL‐6.EVs (DB.EV). (D) Transmission electron microscopy for the WT.EVs and the engineered EVs, scale bar indicates 1 µm.

Firstly, we confirmed that the WT.EVs and the bioengineered EVs possessed the canonical tetraspanin EV surface markers CD9, CD63, and CD81 (Figure 1A), and the intravascular protein TSG101, but did not express calnexin (Figure 1B), nor albumin (Figure S1). confirming purity. In addition, the bioengineered EVs displayed RVG, IL‐6, or both on their surface for RVG.EV, IL‐6.EV and DB.EV respectively (Figure 1A). All EVs showed similar size profiles assessed through NTA and TEM (Figure 1C,D). The bioengineering of the EVs was therefore successful and did not alter the relative size or the expression of canonical proteins compared to WT.EVs.

**FIGURE 2:**
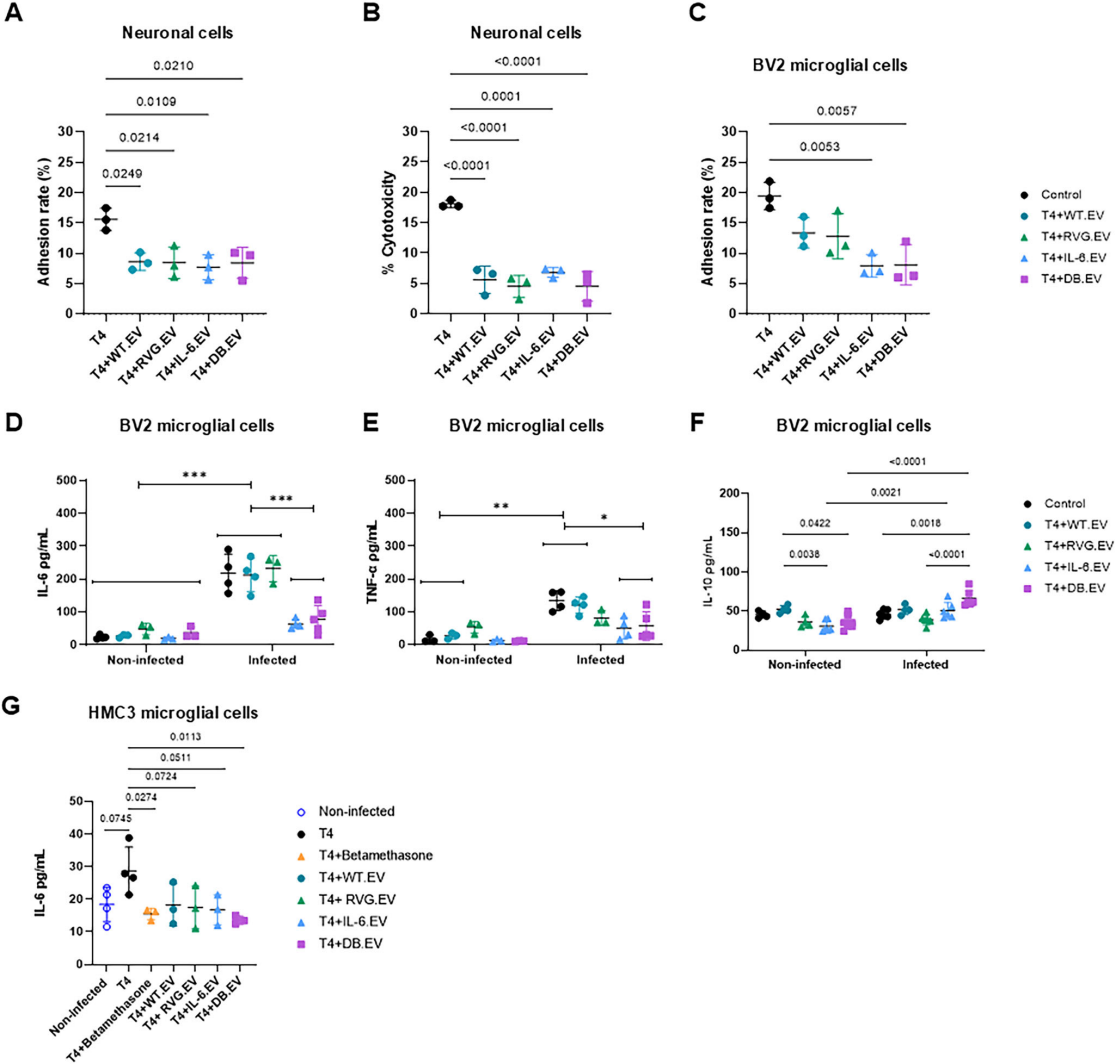
Treatment with EVs during *S. pneumoniae* infection had neuroprotective effects and reduced the release of pro‐inflammatory cytokines. Differentiated SH‐SY5Y neuronal‐like cells infected with *S. pneumoniae* were treated with WT.EVs and bioengineered EVs, this treatment reduced *S. pneumoniae* adhesion rate (A) and cytotoxicity measured by LDH release (B). Treatment also reduced the adhesion of *S. pneumoniae* to BV‐2 microglial cells (C). IL‐6.EV and DB.EV treatment reduced the release of IL‐6 (D) and TNF‐α (E), while the release of IL‐10 was increased after DB.EV treatment (F) during infection of BV2 microglial cells; treatment with all EV types reduced IL‐6 release in HMC3 microglial cells, at a similar degree of betamethasone treatment (G). For A‐C *n* = 3, for D–F *n* = 3‐5, for G *n* = 3–4; the data were analyzed using 2‐way ANOVA with Bonferroni correction for multiple comparisons. Bars depict mean ± SD.

To assess the potential of the EVs to block *S. pneumoniae* interaction with neurons, we used retinoic acid‐differentiated SH‐SY5Y cells as *in vitro* neuronal cell model (Encinas et al. 2000; Hartley et al. 1996; Targett et al. 2024) we infected differentiated human SH‐SY5Y neuronal cells, upon assessment proper cell maturation into neuronal‐like cells (Figure S2), with T4 alone, in combination with WT.EV, RVG.EV, IL‐6.EV and DB.EV, or with respective EV treatment applied 30 min post‐infection. Presence of all EVs led to a significant reduction of bacterial adhesion (Figure 2A) and further reduced neuronal cytotoxicity (Figure 2B). Notably, additional dose‐response experiments were performed using WT.EVs in human neuronal cells; all tested EV concentrations exerted comparable neuronal protection against bacterial adhesion, with no significant differences observed between doses (Figure S3). Imaging revealed that the EVs tethered the plasma membrane of the neurons, a potential protective effect from adhering *S. pneumoniae* (Figure S4A,B). Importantly, treatment with EVs 30 min post‐infection markedly reduced the adhesion of the pneumococcus to neurons. As expected, treatment with the bacteriolytic antibiotic ceftriaxone 30 min post‐infection increased cytotoxicity, a consequence of released intracellular bacterial components, such as Ply. In combination with EV treatment; this pneumococcal‐induced cytotoxicity was reduced but did not reach statistical significance (Figure S5A,B). Taken together, these results indicate that the EVs, regardless of bioengineering, interferes with bacterial components and has neuroprotective effect *in vitro*.

Next, we assessed how the EV treatment would impact inflammation in infected BV‐2 cells, *in vitro* microglial cell model (Henn et al. 2009; Horvath et al. 2008). We observed a reduction in bacterial adhesion upon treatment with EVs, with IL.6.EV and DB.EV treatments led to a significant reduction (Figure 2C), which was retained when EVs were added post‐infection (Figure S5C). Further, we assessed whether the level of cytokine release was altered during treatment with EVs in non‐infected and infected microglia. The IL‐6 levels and TNF‐α levels were markedly reduced during infection for IL‐6.EV and DB.EV‐treated cells (Figure 2D,E). BV‐2 microglial cells upregulate the anti‐inflammatory cytokine IL‐10 in response to LPS and CNS injury (Awada et al. 2014; Dai et al. 2015). Thus, we measured IL‐10 release, which was increased for the DB.EV upon infection (Figure 2F). When applying the EV treatment 30 min post‐infection, no differences were observed in cytokine levels between conditions, however in combination with ceftriaxone treatment IL‐6.EV treatment non‐significantly reduced TNF‐α levels (Figure S5D). These results demonstrate that expression of the signal‐incompetent IL‐6ST decoy receptor mitigated microglial cytokine release during infection.

To provide a clinically relevant anti‐inflammatory reference, we performed IL‐6 quantification from infected HMC3 human microglial cells and treated with EVs, using betamethasone as anti‐inflammatory positive control (Glimåker et al. 2016); our results demonstrate that all EV types induce anti‐inflammatory effects in human microglial cells comparable to those observed following betamethasone treatment, further supporting the immunomodulatory properties of the engineered EVs (Figure 2G).

### 3.2 EVs Sequester Ply, Reducing Its Cytotoxicity

**FIGURE 3:**
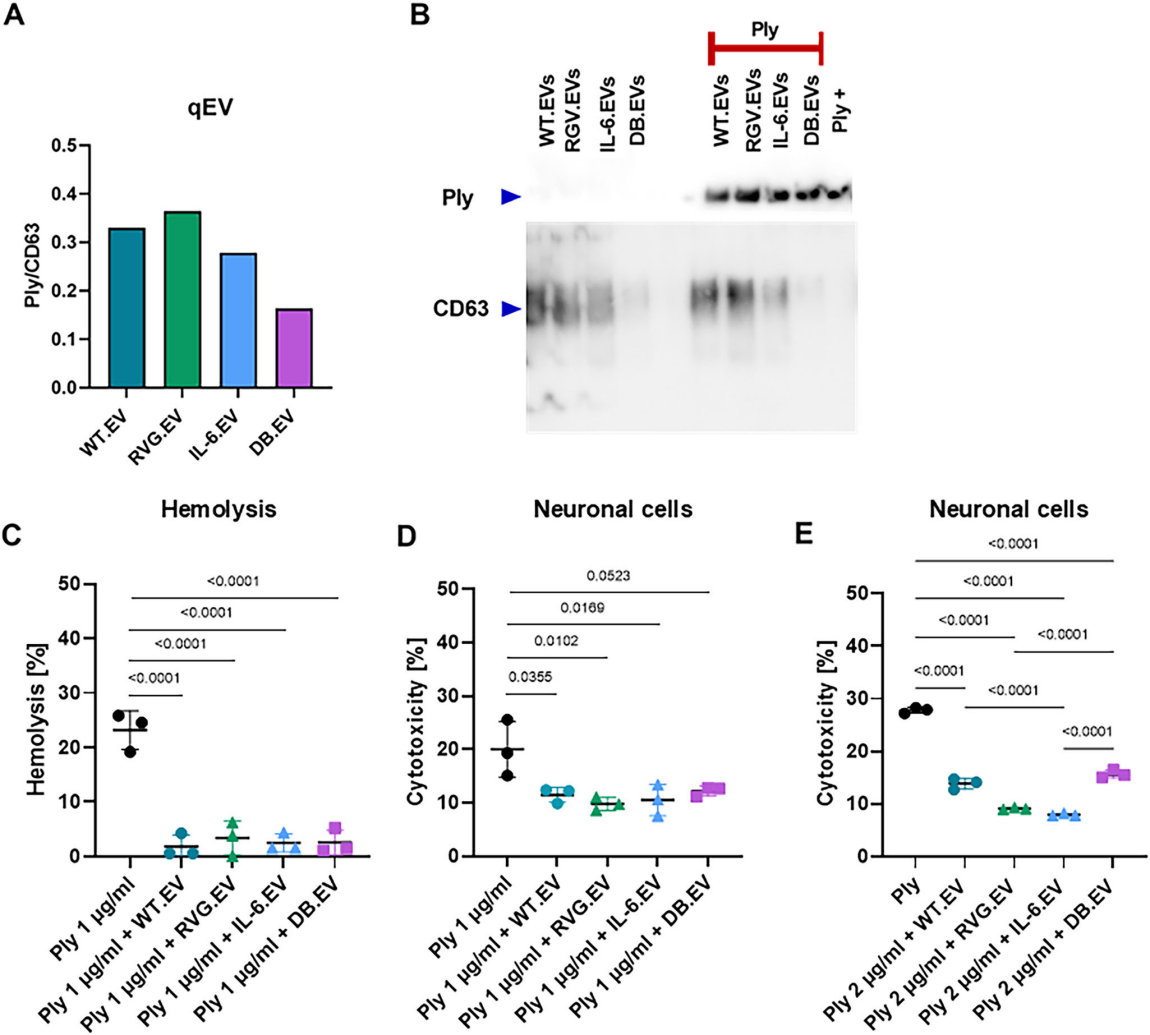
WT.EVs and bioengineered EVs sequestered and neutralized Ply. Ply sequestration was confirmed by incubating 2µg/mL of Ply with 1 × 10^11^ p/mL EV followed by SEC with qEV columns and WB analysis of elute. Quantification demonstrated that all EVs sequestered Ply, graph shows the ratio of band intensity measured by AUC of Ply/CD63 (A) (B) Western blot for Ply and CD63 were used for the quantification. (C) The hemolysis of red blood cells was analyzed upon treatment with Ply 1µg/mL, and the neutralization capacity of the EVs was demonstrated for all EV groups. (D, E) Differentiated SH‐SY5Y neuronal cells were exposed to 1 or 2µg/mL of Ply and treated with 1 × 10^9^ p/mL of respective EVs, which significantly reduced cytotoxicity. qEV experiment was performed once, while *n* = 3 for C–E. The data was analyzed by a one‐way ANOVA, with Bonferroni correction for multiple comparisons. Bars depict mean ± SD.

Given that all EVs exhibited neuroprotective effects in our *in vitro* model and the high content of cholesterol in the EV membrane (Pfrieger and Vitale 2018), we hypothesized that these could sequester Ply and neutralize this toxin. To test this, 2.2 × 10^11^ p/mL EVs were incubated with 2.0 µg/mL of recombinant Ply. All EV types bound Ply (Figure 3A,B), with no difference observed between the size of EVs during incubation (Figure S6). Although not statistically significant, DB.EV depicted a trend towards a higher concentration of Ply in the supernatant, indicating reduced sequestration capacity (Figure S7A,B). We previously reported that Ply facilitates the adhesion of *S. pneumoniae* to neurons by exposing ß‐actin on the neuronal plasma membrane (Tabusi et al. 2021); the capability of EVs to sequester Ply consequently decreases the amount of free Ply that can promote pneumococcal adhesion to neurons leading to a reduced bacterial adhesion in presence of EVs, as shown in Figure 2A. In order to confirm that this sequestering caused a neutralization of the toxin, the hemolytic activity of Ply, which induces lysis of red blood cells at various concentrations (Figure S7C) were assessed upon treatment with different concentrations of WT.EV. WT.EVs reduced 1.0 µg/mL Ply induced lysis at 1 × 10^8^ p/mL (Figure S7D). However, the strongest effect was observed at a concentration 1 × 10^9^ p/mL, and at this concentration all EV groups significantly reduced the hemolytic activity of Ply, confirming their neutralization capacity (Figure 3C). To confirm that this neutralization also protected against Ply‐induced cytotoxicity towards cells, we treated SH‐SY5Y neuronal cells with either 1 µg/mL (Figure 3D) or 2 µg/mL (Figure 3E) Ply in combination with all the EV types and assessed LDH release as a measure of cytotoxicity. All EV types reduced Ply‐induced cytotoxicity towards neurons and demonstrated neuroprotective effects. Next, we sought to evaluate the therapeutic potential of the EVs *in vivo*.

### 3.3 Treatment With Extracellular Vesicles Increased Survival During Experimental Pneumococcal Meningitis

In total four different animal experiments were performed to evaluate the safety and therapeutic effect of the engineered EVs (Table S1). Firstly, to determine if the EVs inhibited *S. pneumoniae* growth *in vivo*, and to confirm that the EVs could enter the brain, we tested three different doses of WT.EVs: 0.5,1, and 2 × 10^11^ p/mouse in our established bacteremia‐derived pneumococcal meningitis model (Farmen et al. 2024; Vander Elst et al. 2025). Mice were infected with *S. pneumoniae* intravenously and received EV treatment one hour later; control animals received PBS. At 8 h post‐infection, no differences in bacterial load in the brain, spleen, or blood were observed between controls and WT.EV treatment groups (Figure S8A). Importantly, we confirmed that the WT.EVs effectively entered the brain parenchyma (Figure S8B). Given the *in vitro* potency of DB.EV treatment, we preliminarily assessed whether a dosage of 2 × 10^11^p/mouse of DB.EVs would have an effect *in vivo*. The mice were sacrificed upon developing severe symptoms of pneumococcal infection. We confirmed that DB.EV treatment significantly improved clinical outcome and increased survival (Figure S8C,D), validating its therapeutic potential and justifying a full‐scale study.

**FIGURE 4:**
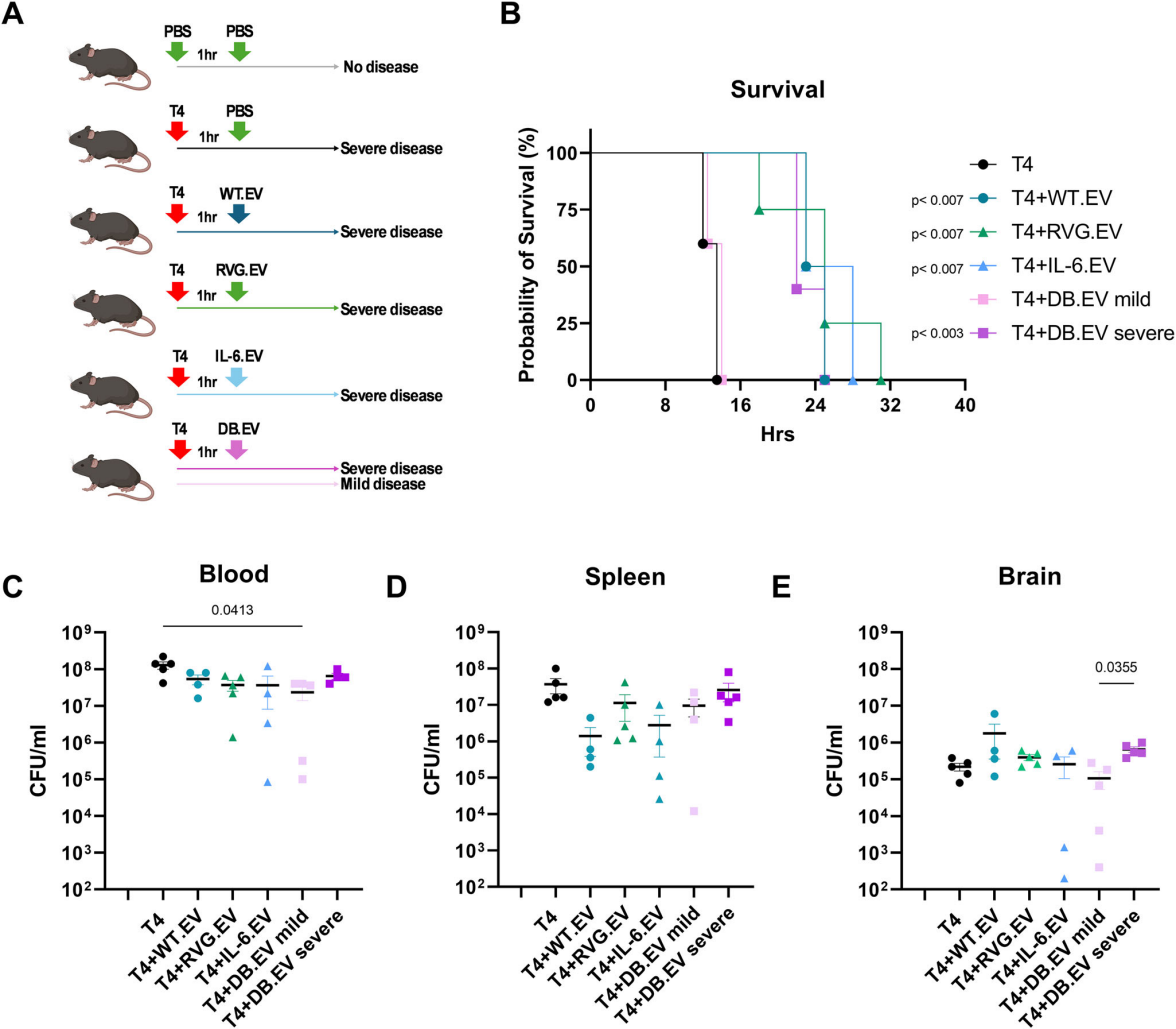
Treatment with EVs increased survival without affecting bacterial loads. Experimental overview of animal groups and treatments (A). Treatment with EVs significantly increased the survival (B). At the time of the experimental endpoint, the animals that reached severe infection showed no significant differences in their bacterial load in the blood (C), spleen (D) or brain (E). DB.EV mild animals showed a slight reduction in bacterial load in the blood and brain. *n* = 4 for WT.EV and IL‐6.EV treated groups, *n* = 5 for T4, RVG.EV, DB.EV for all graphs. Data were analyzed using a one‐way ANOVA and Bonferroni correction for multiple comparisons. Survival curve comparison was tested with the Mantel‐Cox test, the respective p‐value in comparison with T4 (control infected) is annotated next to each group's legend. Bars depict mean ± SD.

Subsequently, all EV treatment groups were evaluated (Figure 4A). In addition, to investigate the early brain pathology and the dynamic inflammatory response observed during pneumococcal meningitis (Barichello et al. 2011), five DB.EV‐treated mice were sacrificed at the same time point as the PBS‐treated infected control group (T4), to minimize time as a confounder in our downstream analysis. The infected control group (T4) had a median time of survival of 13 h; at this time, the DB.EV group presented mild symptoms and was termed “DB.EV mild” (Figure 4B). All other groups were sacrificed when developing severe symptoms of pneumococcal disease.

All EV treatments significantly improved symptomatology and survival, with a median time of survival of 24 h compared to infected controls (T4) (Figure 4B). The bacterial load in the blood (Figure 4C), spleen (Figure 4D), and brain (Figure 4E) did not differ between severe symptom groups; however, the DB.EV mild group had slightly lower bacterial numbers in the blood compared to infected controls (T4), and in the brain compared to the DB.EV severe animal group (Figure 4C). Our results confirmed the therapeutic efficacy of EVs in experimental pneumococcal meningitis, with Ply sequestering as the putative mechanism for the delayed disease onset, due to different EV treatments evoking similar beneficial therapeutic effects. We next assessed if the bioengineered EVs affected the host inflammatory response.

### 3.4 Treatment With RVG.EVs Reduced Inflammation in the Periphery and Brain

The inflammatory response is the main driver of cellular damage in pneumococcal infections, especially in meningitis. Given that the expression of the signal incompetent IL‐6ST decoy receptors reduced the pro‐inflammatory response during infection *in vitro*, we assessed whether treatment with EVs modulated the cytokine release *in vivo*.

**FIGURE 5:**
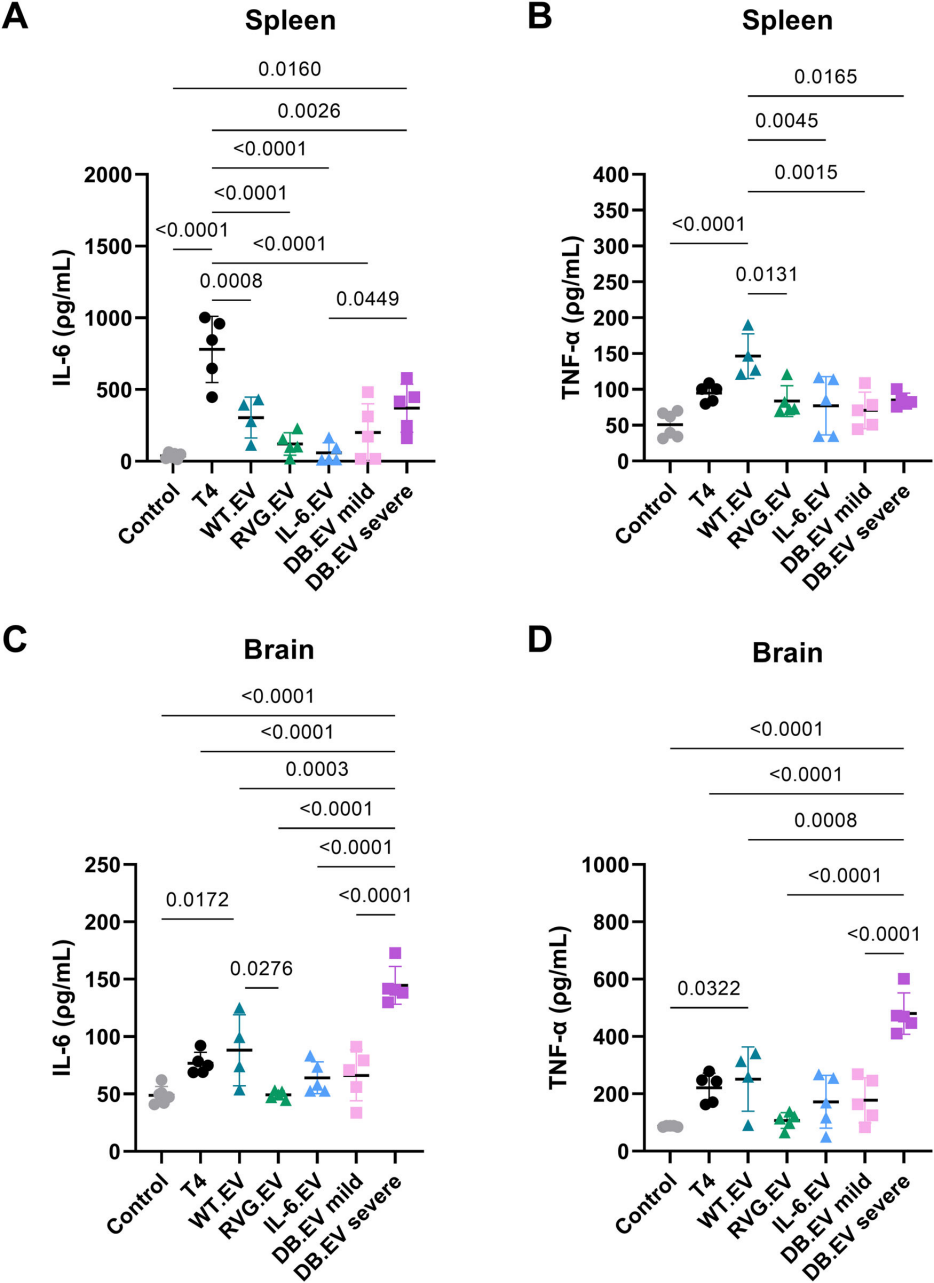
EV treatment altered the release of pro‐inflammatory cytokines in the spleen and brain during infection. In the spleen, the T4 group had the highest levels of IL‐6, however treatment with EVs significantly reduced the release, with the IL‐6.EV group depicting the lowest level (A). TNF‐α was also significantly increased during infection, however treatment with the bioengineered EVs significantly lowered the levels of TNF‐α (B). In brain homogenates, the levels of IL‐6 (C), and TNF‐α (D) were increased during infection for the animal groups with only the RVG.EV depicted lower levels. *n* = 6 for control, *n* = 4 for WT.EV, and *n* = 5 for RVG.EV, IL‐6.EV and DB.EV animal groups. Data were analyzed using a one‐way ANOVA and Bonferroni correction for multiple comparisons. Bars depict mean ± SD.

In the spleen, a crucial component of the peripheral immune system, we observed an increased release of IL‐6 upon infection in the T4 group compared to non‐infected controls (Figure 5A). This increase was significantly reduced for all EV‐treatment groups, with the RVG.EV and IL‐6.EV groups restoring IL‐6 levels close to baseline. TNF‐α release was also elevated upon infection and the WT.EV‐treated animals depicted the highest levels. Importantly, all bioengineered EV treatments reduced the levels of TNF‐α (Figure 5B). Given the systemic nature of the inflammatory response in our intravenous infection‐based mouse model of meningitis, we sought to further validate the peripheral inflammatory profile by western blot analysis. Consistent with the ELISA findings, IL‐6 protein levels in the spleen of infected mice treated with EVs were markedly reduced compared with those in infected control mice that received no EV treatment (Figure S9A,B). Importantly, the concordance between the ELISA and western blot results provides independent evidence that EV administration following infection substantially attenuates the systemic inflammatory response. These findings further support the anti‐inflammatory effect of EV treatment in the context of pneumococcal meningitis.

In the brain, the DB.EV severe group had the highest expression of both IL‐6 and TNF‐α, however, also the WT.EV animals had increased levels of both cytokines (Figure 5C,D). In contrast, the RVG.EV‐treated group had significantly lower expression. Similar findings were also observed in spleen and brain samples from the pilot studies (Figure S10A–E). Additionally, in a separate *in vivo* study, we evaluated the effect of RVG.EV and IL‐6.EV treatment on blood cytokine levels, both treatments reduced IL‐6 and TNF‐α compared to T4 controls, as well as in the spleen and brain (Figure S11A–I). These findings suggest that individual EV treatments modulated inflammation differently, with the RVG.EVs depicting the best neuroprotective effect.

### 3.5 IL‐6ST‐Expressing EVs Modulate Glial Activation Without Affecting Neuronal Survival

**FIGURE 6:**
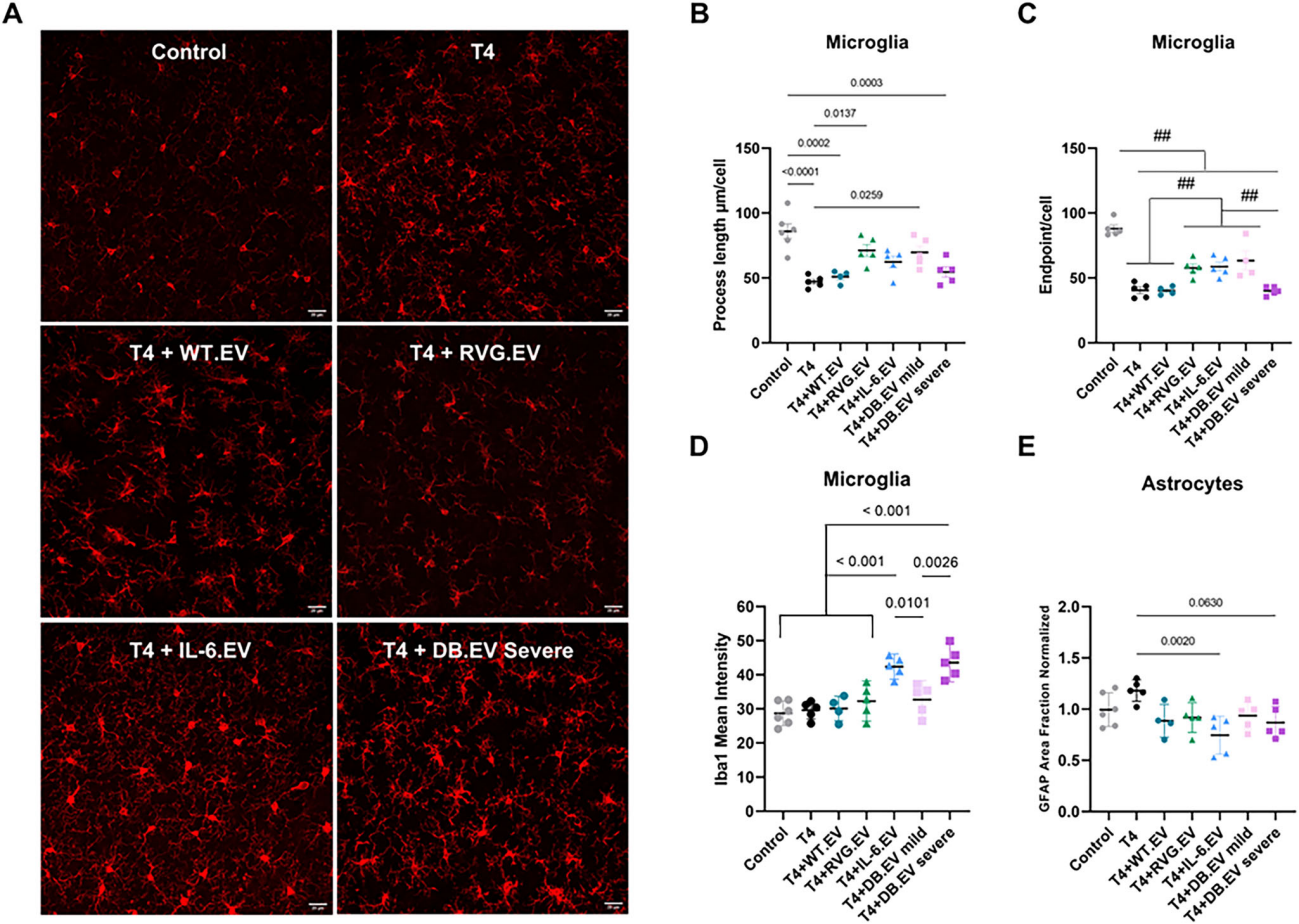
IL‐6ST decoy receptor expression increased microglial activation and reduced GFAP signal, while RVG.EV treatment indicate a protective effect. Representative images from immunofluorescence analysis (A) of microglial cell population (Iba1, red), scalebar depicts 20 µm In‐depth analysis of microglial morphology demonstrated that T4 infection reduced microglial process length (B) and endpoints, ## indicate statistical difference between groups (*p*‐values depicted in Table S2) (C), with different EV treatment affecting both processes. The mean intensity signal of Iba1 (D) and GFAP area coverage (E) were significantly increased for IL‐6.EV and DB.EV animal groups compared with controls and the other infection groups. *n* = 6 for control, *n* = 4 for WT.EV, and *n* = 5 for RVG.EV, IL‐6.EV and DB.EV animal groups. Data was analyzed using a one‐way ANOVA and Bonferroni correction for multiple comparisons. Bars depict mean ± SD.

To assess if treatment of pneumococcal meningitis with different EVs impacted the cellular response in the brain, we analyzed changes in the microglia, astrocytes, and neuronal population. All infected groups had microglia depicting morphological changes compared to the non‐infected controls (Figure 6A and Figure S12A *upper panel*). Infection decreased both microglial process length (Figure 6B) and endpoints (Figure 6C). However, the RVG.EV and DB.EV mild treated groups, demonstrated a significant increase in process length compared to the T4 control group (Figure 6B), indicating reduced microglial activation. Furthermore, both groups, in addition to the IL‐6.EV‐treated group had significantly increased endpoints compared to the other infected groups (Figure 6C). Interestingly, a significant increase in Iba1 intensity was only observed in the IL‐6.EV and DB.EV severe‐treated group (Figure 6D). In addition, both groups showed a lower area coverage of GFAP signalling, indicating reduced astrocytic coverage and protrusion retraction (Figure S12A *middle panel*, Figure 6E). To assess if there was a difference in the number of neurons in the cortex, we stained with the neuronal cell body marker NeuN (Figure S12A *lower panel*), we observed no statistical differences between groups, however, the WT.EV‐treated animals had a slight trend of reduced numbers of neurons and intensity of staining (Figure S12B,C). In summary, RVG.EV treatment reduced the microglial morphological changes, while the IL‐6 trans‐signalling likely plays a key role in brain cellular communication during infection, and its blocking may disrupt glial function, without though exacerbating neuronal loss during the acute phase of infection.

## 4 Discussion

Bacterial meningitis, frequently caused by the pneumococcus, is a devastating neurological disease, which despite the availability of antimicrobials and pneumococcal vaccines, the incidence remains high (Koelman et al. 2020). Survivors have a high risk of developing long‐term neurological sequelae, which are consequences of neuronal damage caused by neuroinflammation and released bacterial compounds, such as Ply (Mook‐Kanamori et al. 2011; Farmen et al. 2021). The treatment strategy is complex, as patients often suffer from complications, such as sepsis and myocarditis, but also brain abscesses and cerebrovascular disease (Legouy et al. 2024; Weisfelt et al. 2006; Van De Beek et al. 2004). The low capacity of antibiotics to cross the BBB and the often cytolytic nature of these render the brain sensitive to the bacteria and bacterial components, such as Ply, for a prolonged period (Brown et al. 2017). It is therefore crucial to develop new therapeutic strategies that can efficiently reach the brain, reduce neuroinflammation, and protect resident brain cells. In this sense, the sequestering and neutralization of Ply is essential. In this study, we have, for the first time, evaluated the treatment potential of bioengineered EVs in pneumococcal meningitis. We have demonstrated that treatment with EVs increased the survival of the infected mice, primarily by sequestering Ply. In addition, by bioengineering the EVs to express RVG peptides, the protective effect of the EVs was extended to the brain, where the treatment reduced inflammation and demonstrated neuroprotective capacity.

EVs have a high therapeutic potential in pneumococcal meningitis and have already been successfully applied in both patients and experimental animal models for cancer, neurodegenerative disease, and systemic inflammatory disease (Putthanbut et al. 2024; Reed and Escayg 2021). Depending on their source, EVs exhibit differences in their intrinsic properties, including low immunogenicity and high tissue penetration (Du et al. 2023). Further, the ability to engineer EVs to express and carry specific cargo increases their potential as therapeutic agents in a variety of diseases (Wiklander et al. 2019). EVs are fast‐acting molecules, and their translocation to tissue has been observed as early as a few minutes after administration (Gupta et al. 2020). In addition, they have a wide array of biodistribution and can exert their effects in multiple tissues. This wide biodistribution is particularly valuable in pneumococcal meningitis, where bacteria frequently invade peripheral organs (Weiser et al. 2018; Wiklander et al. 2015). The crossing of neural barriers by native EVs involves transcytosis, and while no direct evidence is observed in humans, several *in vitro* models have demonstrated that EVs cross endothelial cell barriers in an inflammatory milieu (Morad et al. 2019; Banks et al. 2020). While using a human endothelial‐cell monolayer can provide useful preliminary insights into drug delivery across the BBB, *in vitro* cellular systems remain an artificial model that lacks the full complexity and physiological context of the human BBB (Yang et al. 2023). Thus, *in vivo* BBB passage data are essential to validate delivery strategies, meaning findings in animal models, such as the bacteremia‐derived meningitis model used in this study. In pneumococcal meningitis patients, bacterial components and the systemic immune response cause significant vascular changes in the brain, including BBB disruption, facilitating an increase in antibiotic infiltration into the brain (Engelen‐Lee et al. 2016; Yang et al. 2023). However, treatment with steroids, such as dexamethasone in the case of pneumococcal meningitis, can reduce CSF antibiotic concentrations by limiting BBB permeability (Haddad et al. 2022). In both animal models and patients a disruption of the BBB, to a variable degree, is observed (Sharief et al. 1992; Barichello et al. 2012), nevertheless EVs bioengineered with RVG peptides have a therapeutic advantage as they can cross human endothelial cell layers through receptor‐mediated endocytosis, regardless of the BBB permeability due to the presence of AchR on the BBB, and suggest that these EVs could be promising for clinical applications (Wiklander et al. 2015; Kumar et al. 2007).

During pneumococcal meningitis, host cells are exposed to the cholesterol‐binding cytolysin Ply, which causes a plethora of harmful effects on the host, including cell lysis, activation of pro‐inflammatory cascades, and increased production of reactive oxidative species (Nishimoto et al. 2020; Mitchell and Dalziel 2014). The neutralization of Ply during infection is crucial for reducing neuronal damage and, consequently, the onset of neurological sequelae in patients (Nishimoto et al. 2020; Cima Cabal et al. 2023). Previous studies using liposomes have demonstrated the interaction between cholesterol and Ply, with an increase in cholesterol content associated with increased sequestration. Similar to liposomes, the EV membrane contains cholesterol (Pfrieger and Vitale 2018), and in our study, the EVs sequestered and neutralized Ply, which protected against Ply‐induced neurotoxicity. Importantly, during this sequestration, the abundance of circulating Ply targeting host cells is reduced, conferring a neuroprotective effect *in vitro*. *In vivo*, this ultimately mitigated both cytotoxicity and inflammation and delayed disease onset. This treatment benefit seems primarily driven by the peripheral sequestration of Ply as all EVs delayed disease onset, while RVG.EVs additionally conferred protection within the brain. Given that 81% of pneumococcal meningitis patients have positive blood cultures for *S. pneumoniae* and 39% of deaths are attributed to systemic complications, this peripheral effect may have therapeutic relevance (Sharew et al. 2020; Koelman et al. 2022). Furthermore, in cases of prolonged bacterial presence in the brain, either due to limited BBB penetration of antibiotics or antibiotic‐resistant *S. pneumoniae*, or the use of bacteriolytic antibiotics that exacerbate brain injury (Grandgirard et al. 2007), we hypothesize that RVG.EVs, owing to their enhanced brain infiltration, could provide added therapeutic benefit.

Ply also induces a pro‐inflammatory response and contributes to the peripheral and neuroinflammatory process during pneumococcal meningitis; the latter is the main cause of neurological sequelae in patients (Wall et al. 2021; Hupp et al. 2022). Microglia, together with other brain resident cells, recognize bacterial compounds inducing upregulation of the NF‐κβ pathway and the release of pro‐inflammatory cytokines (Mook‐Kanamori et al. 2011). Dexamethasone treatment reduces inflammation in patients and has been reported to improve outcomes; however, animal studies indicate that an inflammatory response promotes survival (De Gans and Van De Beek 2002; Albrecht et al. 2016). There exists a very delicate balance between beneficial and harmful inflammatory responses during pneumococcal meningitis; an inadequate response induces a higher risk of tissue damage (Gerber et al. 2004; Koedel et al. 2004; Echchannaoui et al. 2002), while prolonged inflammation increases tissue damage and risk of death (Mook‐Kanamori et al. 2011). Increased levels of TNF‐α and IL‐6 correlate with tissue damage in animal models, and these cytokines are markedly increased in the CSF of bacterial meningitis patients (Barichello et al. 2010; Coutinho et al. 2013). IL‐6 is produced in response to infection and cellular damage and exerts its pro‐inflammatory effects by binding IL‐6ST receptors ubiquitously expressed, termed the trans‐signalling pathway (Rose‐John et al. 2017). Treatment with EVs expressing signal incompetent IL‐6ST decoy receptor reduced LPS‐induced systemic inflammation and neurodegenerative disease pathogenesis (Gupta et al. 2021; Cheng et al. 2023). In our study, EVs expressing signal‐incompetent IL‐6ST decoy receptors (IL‐6.EVs) reduced peripheral IL‐6 levels yet failed to dampen inflammation in the brain. Moreover, they induced increased microglial activation and astrocytic process retraction. We can only hypothesize that the trans‐signalling pathway of IL‐6 plays an important role in cellular communication in the brain during infection, and its absence alters the function of glial populations. However, the effect on the glial populations did not cause neuronal damage.

Interestingly, treatment with RVG.EV significantly lowered TNF‐α and IL‐6 levels in the brain and the periphery. While the protective effect of the RVG.EV treatment in the brain could be explained by its higher affinity for brain infiltration, where they neutralize and sequester Ply; the anti‐inflammatory effect in the spleen and blood was not expected. The spleen has the second‐highest uptake of EVs in the body after the liver, and it might cause an increased Ply sequestration and reduced inflammation (Gupta et al. 2023). Additionally, the spleen, while not directly innervated by the vagus nerve, plays a crucial role in the systemic cholinergic anti‐inflammatory pathway through the splenic nerve and abundance of Ach receptors (Fatemikia et al. 2022). The binding of Ach to these receptors has an inhibitory effect on inflammation (Wu et al. 2021). Although the RVG peptide has been demonstrated to be a competitive antagonist using the *Xenopus laevis* oocyte model, it is to our knowledge no evidence on the effect of the RVG peptide on immune cells (O'Brien et al. 2024). The overall anti‐inflammatory effect of RVG.EV treatment in the *in vivo* study was not observed for the microglial cell line, highlighting the complexity of the immune response during pneumococcal meningitis.

In this study, we have exclusively focused on EV treatment in pneumococcal meningitis, which is the most severe form of pneumococcal disease. The neutralization of Ply by the EVs will also apply to other pneumococcal infections, such as myocarditis and pneumonia, where Ply contributes to the damage on cardiac and lung cells, respectively (Alhamdi et al. 2015; Rai et al. 2016). Our results support previous research using liposomes, that neutralization of Ply is key to the successful treatment of pneumococcal infections (Besançon et al. 2021; Müller et al. 2023; Watt et al. 2025). Additionally, cholesterol‐binding cytolysin production is not a unique feature of the pneumococcus; other meningitis‐causing bacteria also produce this type of toxin, including *Streptococcus pyogenes*, which produces Streptolysin O (Pramitasuri et al. 2023). Ultimately, using EVs to reduce toxicity can also be extended to these infections. The therapeutic potential of EVs, liposomes, and other Ply neutralizing agents is especially relevant in combination with bacteriolytic antibiotics. These antibiotics cause bacterial lysis, augmenting Ply release and increasing tissue damage (Grandgirard et al. 2007). Although not statistically significant, our *in vitro* data, when employing such a combination treatment, did indicate a reduction in both cytotoxicity and inflammation. Nevertheless, future studies combining EV treatment with different antibiotic classes and steroids, *in vitro* and *in vivo*, is needed to confirm its safety and potential beneficial effect. Also relevant for the potential therapeutic feasibility of the RVG.EVs are the potential adverse effect of the RVG peptides on cholinergic neurons. The peptide is crucial for uptake of the rabies virus into neurons and can influence neuronal excitability and signaling; causing behavioral changes in model systems (O'Brien et al. 2024; Hueffer et al. 2017). However, RVG exosome treatment remains a promising strategy for targeting CNS disease, with previous studies demonstrating no adverse effect of the treatment (Alvarez‐Erviti et al. 2011). Multiple preclinical studies engineered exosomes with RVG (Lamp2b–RVG or RVG29 surface display) to deliver siRNA or therapeutic cargos to the brain after systemic delivery; these studies reported efficient neuron targeting and gene knockdown and observed no non‐specific uptake or attenuation on repeat dosing (no overt adverse effects), and subsequent preclinical work has reproduced effective brain delivery and therapeutic benefit without obvious acute toxicity (Alvarez‐Erviti et al. 2011; Liu et al. 2015).

One important aspect to consider is that we evaluated EVs only during the acute phase of meningitis and as a standalone treatment *in vivo*. The bacteremia‐derived mouse model, while reflective of natural infection routes, also induces sepsis, unlike models using direct CSF inoculation (Intracisternal (I.C) model) combined with antibiotics (Grandgirard et al. 2007). Nevertheless, the peripheral immune system and tissues are also involved in disease progression in most cases of pneumococcal meningitis (Weiser et al. 2018). Notably, the reduction in peripheral IL‐6 following EV treatment was independently confirmed by ELISA and Western blot, further highlighting the contribution of systemic inflammation to our disease model. In contrast to I.C models, the animals are sacrificed at an earlier stage in the bacteremia‐derived model; thus, there are fewer neuropathological features (Chiavolini et al. 2008). Furthermore, although EV treatment improved survival and reduced inflammatory markers, the present study did not evaluate intracranial pressure (Lindvall et al. 2004), which is an important contributor to mortality in acute bacterial meningitis. Another limitation of our study is the use of an animal model in which all mice succumb to infection; thus, whether the therapeutic effect is maintained across varying degrees of infection severity is yet to be determined. Future studies are needed to confirm the effect of such treatment in a milder infection scenario, and its safety and protective effect in combination with antibiotics. While all EVs protected against pneumococcal interaction and Ply cytotoxicity *in vitro*, our *in vivo* results did not demonstrate a significant neuroprotective effect, potentially due to the high systemic disease burden in our animal model. Although we clearly showed that, *in vitro*, EVs can protect neuronal cells from bacterial interaction and Ply‐induced cytotoxicity.

In conclusion, our results strongly underline the importance of Ply neutralization in pneumococcal meningitis and the effectiveness of EVs in this manner. Our results indicate that expression of RVG peptide and signal incompetent IL‐6ST decoy receptor on EVs had a substantial benefit as it (i) suppressed peripheral inflammation, (ii) increased the EVs capacity to reach the brain, where they (iii) sequestered Ply (iv) potentially providing neuronal protection from toxin‐induced injury, and (v) lowering excessive neuroinflammation. Taken together, RVG‐expressing EVs present a compelling adjuvant strategy for future treatment of pneumococcal meningitis.

## Author Contributions

**Kristine Farmen**: conceptualization, investigation, writing – original draft, methodology, validation, visualization, writing – review and editing, formal analysis, data curation, resources. **Samir El Andaloussi**: conceptualization, investigation, writing – review and editing, resources, data curation, funding acquisition. **Xiuming Liang**: investigation, methodology, validation, writing – review and editing, formal analysis, data curation, resources. **Federico Iovino**: conceptualization, investigation, funding acquisition, writing – original draft, methodology, validation, visualization, writing – review and editing, formal analysis, project administration, data curation, supervision, resources. **Miguel Tofiño‐vian**: conceptualization, investigation, writing – review and editing, methodology, data curation, resources. **Oscar P. B. Wiklander**: conceptualization, investigation, funding acquisition, writing – review and editing, data curation, resources, project administration, supervision. **Georgia Yfanti**: investigation, writing – review and editing, methodology, validation, data curation, resources. **Houze Zhou**: investigation, methodology, validation, writing – review and editing, formal analysis, data curation, resources. **Doste R. Mamand**: investigation, writing – review and editing, methodology, validation, formal analysis, data curation, resources. **Vicky W. Q. Hou**: investigation, methodology, validation, writing – review and editing, formal analysis, data curation, resources.

## Funding

This research was funded by grants, held by FI, from The Swedish Research Council (Vetenskapsrådet) (2020‐02061), Bjarne Ahlström Memorial Fund, the European Society for Clinical Microbiology and Infectious Diseases (ESCMID), The Strategic Research Area Neuroscience at Karolinska Institutet, Umeå University and KTH (StratNeuro), Clas Groschinsky Foundation, HKH Crown Princess Lovisa's Association for Children's Healthcare, Karolinska Institutet, Magnus Bergvall Foundation, Ålhén Foundation, Wera Ekström Fund for Pediatric Research, Jane och Dan Olsson Foundation, and Tysta Skolan Foundation. OPBW is supported by: The Swedish Research Council (VR, 2022–02449), the Center for Innovative Medicine (CIMED, FoUI‐1024690), the Swedish Cancer Society (Cancerfonden, 23 2935 Pj 01 H), the Cancer Research Foundations of Radiumhemmet (Rahfo, 241392), the Swedish Brain Foundation (Hjärnfonden, FO2025‐0365), and Karolinska Institutet (2‐116/2023). SEA is supported by the European Research Council (ERC) under the European Union's Horizon 2020 research and innovation program (DELIVER, grant agreement No. 101001374), Swedish Research Council (4‐2538/2025), Brain foundation (Hjärnfonden) contract (FO2024‐0073‐TK‐113), and CIMED.

## Conflicts of Interest

Oscar P. B. Wiklander and Samir El‐Andaloussi hold stock interests in Evox Therapeutics. Samir El‐Andaloussi is also the founder. The other authors declare no competing interests.

## Supporting information




**Supporting information**: jev270369‐sup‐0001‐SuppMat.docx

## Data Availability

All data generated and analyzed in this study are included or can be provided upon request.
