# Supplementary material for: Bioengineered Extracellular Vesicles Mitigate Neuroinflammation by Neutralizing Pneumolysin and Delaying Disease Onset in Experimental Pneumococcal Meningitis
Source: J Extracell Vesicles. 2026 Sep 11;15(9):e70369. doi: 10.1002/jev2.70369 (PMC13563070; doi:10.1002/jev2.70369)
Supplement: Supplementary file 1 [file JEV2-15-e70369-s001.docx]

**Bioengineered extracellular vesicles mitigate neuroinflammation by neutralizing pneumolysin, delaying disease onset in experimental pneumococcal meningitis**

Kristine Farmen^1^, Doste R. Mamand^2,3,4,5^, Miguel Tofiño-Vian^1^, Georgia Yfanti^1^, Xiuming Liang^2,3,6^, Houze Zhou^2,3,6^, Vicky W.Q. Hou^2,3,6^, Samir El Andaloussi^2,3,6^, Oscar P. B. Wiklander^2,3,4^, and Federico Iovino^1^

^1^Department of Neuroscience, Karolinska Institutet, Stockholm, Sweden

^2^Division of Biomolecular and Cellular Medicine, Department of Laboratory Medicine, Karolinska Institutet, Stockholm, Sweden

^3^Karolinska ATMP Center, Karolinska Institutet, Stockholm 14152, Sweden

^4^Breast Center, Karolinska Comprehensive Cancer Center, Karolinska University Hospital, Stockholm, Sweden

^5^Biology Research center, Research Center, University of Zakho, Zakho, 42002, Duhok, Kurdistan Region, Iraq

^6^Department of Cellular Therapy and Allogeneic Stem Cell Transplantation (CAST), Karolinska University Hospital, Stockholm, Sweden

Correspondence: Federico Iovino, [federico.iovino@ki.se](mailto:federico.iovino@ki.se)

**Supplementary material**

| **Study** | **1** | **2** | **3** | **4** |
| --- | --- | --- | --- | --- |
| **Type** | Pilot | Pilot | Full-scale | Add-on |
| **Purpose** | - Confirm dosage safety and titration - *S. pneumoniae* growth in presence of EVs - EV crossing of the BBB | - Assess DB.EV effect on survival | Assess EV treatment effect on:   - Survival - CFU - Inflammation - Neuroprotection | Assess EV treatment effect on:   - Blood cytokine levels - Spleen cytokine levels - Brain cytokine levels |
| **Mice (n)** | 3 each group | 4-7 | 3-5 | 3 |
| **EV type** | WT.EV (termed mild in Supp Fig 7) | DB.EV | WT.EV, RVG.EV, IL-6.EV, DB.EV | RVG.EV, IL-6.EV |
| **Endpoint and tissue collection** | 8 hours post-infection | Score of 0.4/0.5*DB.EV mild sacrificed together with T4 control | Score of 0.4/0.5*  *DB.EV mild sacrificed together with T4 control | Score of 0.4/0.5 |
| **Downstream analysis** | CFU, WB for CD81,  cytokine release | Survival, symptoms,  cytokine release | CFU, cytokine release, confocal analysis | Cytokine release |
| **Figure** | Supplementary Figure 6A.B and 7 | Supplementary Figure 6,7C.D and 9. | Figure 4-6, and Supplementary Figure 9 | Supplementary Figure 8 |

**Supplementary Table 1.** Overview of the four *in vivo* studies included, their purpose, n-number, type of EV used, endpoint and tissue collection, downstream analysis and the figure presenting the results.

| **Comparison** | **p-value** |
| --- | --- |
| Control vs. T4 | <0.0001 |
| Control vs. T4+WT.EV | <0.0001 |
| Control vs. T4+RVG.EV | <0.0001 |
| Control vs. T4+IL-6.EV | <0.0001 |
| Control vs. T4+DB.EV mild | 0.0007 |
| Control vs. T4+DB.EV severe | <0.0001 |
| T4 vs. T4+RVG.EV | 0.0212 |
| T4 vs. T4+IL-6.EV | 0.0117 |
| T4 vs. T4+DB.EV mild | 0.0019 |
| T4+WT.EV vs. T4+RVG.EV | 0.0316 |
| T4+WT.EV vs. T4+IL-6.EV | 0.0181 |
| T4+WT.EV vs. T4+DB.EV mild | 0.0031 |
| T4+RVG.EV vs. T4+DB.EV severe | 0.0164 |
| T4+IL-6.EV vs. T4+DB.EV severe | 0.009 |
| T4+DB.EV mild vs. T4+DB.EV severe | 0.0015 |

**Supplementary Table 2.** P-values for Figure 6C (microglial endpoints).

**Supplementary Figure 1. Assessment of EV purity by western blot for albumin. (A)** Coomassie staining depicting the EVs, media (no-serum included), serum diluted and non-diluted. (**B)** Western blot for albumin.

**Supplementary Figure 2. Differentiation of SH-SY5Y cells into mature neuronal cells.** Cell lysates of SH-SY5Y cells at different time-points (4, 7, 9 and 11 days) after retinoic acid treatment were used for western blot analysis for MAP2 detection; with the monoclonal anti-MAP2 antibody sc-74421, two MAP2 bands are detected at 45 and 65 kDa. The band intensity increase from 4 to 11 days demonstrates the successful differentiation of SH-SY5Y cells.

**Supplementary Figure 3. Dose titration of WT.EV treatment on infected neurons.** Adhesion of *S. pneumoniae* T4 strain on human neuronal cells was quantified in absence of EV treatment (Positive), and under several concentrations of WT.EV, non-infected cells were used as negative control; n=2-5, bars depict mean ± SD.

**Supplementary Figure 4. EVs on neuronal cells.** Representative images from SEM showing control infected (T4), and infected + DB.EV treatment of SH-SY5Y neuronal-like cells (**A**). ***Upper panel*** indicates low magnification; red squares indicate the area of higher magnification in the ***lower panel****.* Red arrows indicate *S. pneumoniae*. Green arrows indicate DB.EV clusters. Scalebar indicate 50μm The DB.EV was also observed targeting the SH-SY5Y neuronal cell body. representative image depicts confocal imaging (**B**). The white arrows indicate the EVs represented as bright green circles.

**Supplementary Figure 5. Treatment with EVs post *S. pneumoniae* infection with and without antibiotics demonstrated neuroprotective effects.** Differentiated SH-SY5Y neuronal-like cells infected with *S. pneumoniae* were treated with WT.EVs and bioengineered EVs 30 min post-infection, this treatment reduced *S. pneumoniae* adhesion rate (**A**). In combination with 25μg/ml Ceftriaxone (Cfx), the EV treatment demonstrated a trend in reducing cytotoxicity measured by LDH release (**B**). Treatment also reduced the adhesion of *S. pneumoniae* to BV-2 microglial cells when added 30-min post infection (**C**). In combination with 25μg/ml Cfx, the EV treatment demonstrated trend in reducing IL-6.EV treatment demonstrated a trend in reducing TNF-α levels (**D**). For A-D, n=2-4 for all treatment groups, data were analysed with a one-way ANOVA and Bonferroni post-hoc test for multiple comparisons. The data were analysed using 2-way ANOVA with Bonferroni correction for multiple comparisons. Bars depict mean ± SD.

**Supplementary Figure 6. NTA from Ply-sequestering experiments.** The interaction between Ply and the EVs was assessed through size exclusion chromatography (qEV). The eluate was collected and assessed with WB (Figure 3A, B) and NTA was performed on the sample, the ***upper panel*** depicts the size of the EVs without Ply, and the ***lower panel*** depicts the size of the EVs with Ply.

**Supplementary Figure 7. Sequestration of Ply by EVs and titration of Ply and EV dosage in hemolytic assay.** The Ply concentration in the pelleted and supernatant fractions from ultracentrifugation of EV-Ply mixture was assessed by WB (**A, B**). Dose-titration of recombinant Ply concentration to induce hemolysis (**C**). Three different WT.EV concentrations were used to assess their sequestering of Ply; incubation time was 45min (**D**). Experiments were performed in triplicates. Bars depict mean ± SD.

**Supplementary Figure 8. Pilot studies.** Animals were infected with *S. pneumoniae* I.V. then one hour later received EV treatment I.V. Dose titration of the EVs showed that there were no significant differences between bacterial load in the periphery and brain between animal groups (**A**). From brain homogenates we performed western blot analysis for the human CD81 protein. showing that the generated EVs were only in the brain of EV-treated animals and present at 8hrs post-infection (**B**). Assessment of the effectiveness and safety of the DB.EV showed that survival was significantly increased in the DB.EV treated group compared to T4 animal group (**C**). with the majority of DB.EV showed absence or mild symptoms at 30hrs while T4 animal group depicted severe symptoms (**D**). Bars depict mean ± SD.

**Supplementary Figure 9. Peripheral inflammation during infection with and without EV treatment assessed by western blot analysis.** Detection of IL-6 by western blot using spleen homogenates from mice infected with T4, with or without EV treatment; Coomassie staining of SAS-page gel with the same samples was used as loading control (**A**); IL-6 band intensity was quantified and normalized based on the total protein content quantified using the Coomassie staining (**B**); n= 3, data were analysed using a one-way ANOVA and Bonferroni correction for multiple comparisons. Bars depict mean ± SD.

**Supplementary Figure 10. Effect of engineered EV treatment on release of cytokines from pilot studies,** Animals were infected with *S. pneumoniae* I.V. then one hour later received EV treatment I.V. WT.EV group was sacrificed 8-hrs post infection and termed WT.EVmild, while DB.EV mild sacrificed together with T4 infected. DB.EV severe and T4 were sacrificed upon developing clinical symptoms of severe disease. Between animal groups there were no significant differences in bacterial load in the blood (**A**) that could influence the cytokine levels. In the spleen the infection increased both IL-6 (**B**) and TNF-α (**C**), the latter was significantly reduced in WT.EV mild and DB.EV mild animal groups. In the brain, both groups depicted reduced IL-6 (**D**) and TNF-α (**E**), with DB.EV severe demonstrated the highest level of IL-6 as observed in the main study (Figure 5). n= 6 for control, n =3 for T4 and EV treatment groups. Data were analysed using a one-way ANOVA and Bonferroni correction for multiple comparisons. Bars depict mean ± SD.

**Supplementary Figure 11. The correlation of cytokine levels in plasma, spleen and brain.** Animals were infected with *S. pneumoniae* I.V. then one hour later received EV treatment I.V. CFU quantification showed that there was a slight increase in bacterial load in the blood (**A**), spleen (**B**) and brain (**C**) in RVG.EV-treated animals compared to T4 control and IL-6.EV. Despite this both RVG.EV treatment and IL-6.EV treatment reduced levels of IL-6 in plasma (**D**), spleen (**E**), and brain (**F**). TNF-α was non-significantly reduced in plasma (**G**) but was reduced in the brain and spleen for both EV treatment groups (**H**) and (**I**), n= 6 for control and n = 3 for T4 and EV treatment groups. Data were analysed using a one-way ANOVA and Bonferroni correction for multiple comparisons. Bars depict mean ± SD.

**Supplementary Figure 12. Assessment of neuropathology in pneumococcal meningitis.** Representative images from immunofluorescence analysis (**A**) of microglial cell population (Iba1, red) ***upper panel****,* astrocytes (GFAP, green) and blood vessels (lectin, red) ***middle panel****,* and neurons (NeuN, green) ***lower panel***. No significant difference was observed for the total number of neurons in the F.O.V (**B**) nor intensity of staining (**C**) between animal groups, although WT.EV showed a slight trend in reduction. n= 3 for control, n=4 for WT.EV, and n=5 for RVG.EV, IL-6.EV and DB.EV animal groups. Data were analysed using a one-way ANOVA and Bonferroni correction for multiple comparisons. Bars depict mean ± SD. Scalebar indicate 20μm.
